# Plastome phylogeny and lineage diversification of Salicaceae with focus on poplars and willows

**DOI:** 10.1002/ece3.4261

**Published:** 2018-07-13

**Authors:** Lei Zhang, Zhenxiang Xi, Mingcheng Wang, Xinyi Guo, Tao Ma

**Affiliations:** ^1^ Key Laboratory of Bio‐Resource and Eco‐Environment of Ministry of Education College of Life Sciences Sichuan University Chengdu Sichuan China

**Keywords:** chloroplast genome, gene loss, phylogenetic relationship, Salicaceae *sensu lato*

## Abstract

Phylogenetic relationships and lineage diversification of the family Salicaceae *sensu lato* (*s.l*.) remain poorly understood. In this study, we examined phylogenetic relationships between 42 species from six genera based on the complete plastomes. Phylogenetic analyses of 77 protein coding genes of the plastomes produced good resolution of the interrelationships among most sampled species and the recovered clades. Of the sampled genera from the family, *Flacourtia* was identified as the most basal and the successive clades comprised both *Itoa* and *Poliothyrsis*,* Idesia*, two genera of the Salicaceae *sensu stricto* (*s.s*.) (*Populus* and *Salix*). Five major subclades were recovered within the *Populus* clade. These subclades and their interrelationships are largely inconsistent with morphological classifications and molecular phylogeny based on nuclear internal transcribed spacer sequence variations. Two major subclades were identified for the *Salix* clade. Molecular dating suggested that species diversification of the major subclades in the *Populus* and *Salix* clades occurred mainly within the recent Pliocene. In addition, we found that the *rpl32* gene was lost and the *rps7* gene evolved into a pseudogene multiple times in the sampled genera of the Salicaceae *s.l*. Compared with previous studies, our results provide a well‐resolved phylogeny from the perspective of the plastomes.

## 1 INTRODUCTION

The family Salicaceae *sensu lato* (*s.l*.) contains more than 50 genera and 1,000 species (Chase, Zmarzty, Lledó, & Wurdack, 2002), although the formerly defined Salicaceae *sensu stricto* (*s.s*.) only comprises two genera, *Salix* and *Populus* (Fang, Zhao, & Skvortsov, 1999; Ohashi, 2001). The woody species in the Salicaceae *s.l*., ranging in height from less than few centimeters to tens of meters, are found from the Arctic to the equator and occupy extremely varied habitats (Chase et al., 2002). The sexual systems employed by this family are highly diverse. Most genera are dioecious but some are monoecious. However, both XY and ZW sex determination systems have been reported in dioecious species (Hou et al., 2015; Kersten, Pakull, Groppe, Lueneburg, & Fladung, 2014), suggesting strikingly dynamic sex determinations through the diversification history. Chemicals produced by the family are varied. For example, the early modern medicine aspirin was first isolated from the bark of willows and poplars, while *Idesia* fruits synthesize an abundant oil containing unsaturated fatty acids (Li et al., 2016). In addition, poplars are among the keystone components of the temperate and boreal forest communities in the North Hemisphere and are widely cultivated worldwide, accounting for more than half of the planted forests in China used for the paper, pulp, and wood industries (Hamzeh & Dayanandan, 2004; Stettler, Bradshaw, Heilman, & Hinckley, 1996). Willows have also been developed as bioenergy crops (Smart & Cameron, 2008). The high diversification with respect to numerous traits and ecologically and economically important applications has attracted large numbers of scientists to use the Salicaceae *s.l*. as a model system for comparative studies of diverse important traits (including reproductive systems, habits, and chemicals) to investigate underlying ecological drivers or genetic mechanisms (Bradshaw, Ceulemans, Davis, & Stettler, 2000; Ellis, Jansson, Strauss, & Tuskan, 2010; Jansson & Douglas, 2007). However, all of these comparisons need a robust phylogenetic framework that provides a robust knowledge of the interrelationships and divergence timescales.

Most previous phylogenetic work focused on the relationships of the main subclades of the genera *Salix* and *Populus* because species delimitation in them remains disputed. In the genus *Populus*, there are six recognized sections (sects. *Abaso*,* Turanga*,* Populus*,* Leucoides*,* Aigeiros*, and *Tacamahaca*) consisting of 29–70 species (Eckenwalder, 1996; Fang et al., 1999). Sectional relationships have not been well resolved or have proved inconsistent based on the sequence variations from both the nuclear internal transcribed spacer (ITS) and several chloroplast DNA regions (Cervera et al., 2005; Hamzeh, Périnet, & Dayanandan, 2006; Wan et al., 2013). Around 450 species have been published for the genus *Salix* and the available phylogenetic studies were carried out mainly based on ITS or several chloroplast DNA regions (Chen, Sun, Wen, & Yang, 2010; Lauron‐Moreau, Pitre, Argus, Labrecque, & Brouillet, 2015; Wu et al., 2015). Two main subclades have been identified. Two recent studies expanded sampling to more genera of the Salicaceae *s.l*. in addition to *Populus* and *Salix* and reconstructed the phylogenetic relationships within the family based on 13 genes from the plastid, mitochondrial and nuclear genomes or chloroplast genomes, respectively (Wurdack & Davis, 2009; Xi et al., 2012). However, these two studies did not sample enough species of the genera *Populus* and *Salix*. Because of this sampling limitation, our understanding of phylogenetic relationships and divergence timescales between the major clades of the family remain unclear. In this study, we resequenced and aligned chloroplast genomes of 28 additional species for the family, focusing on poplars and willows. We mainly aimed to: (a) construct a phylogeny based on the plastomes of 42 species and examine their congruence with morphological delimitation and previous molecular phylogenies based on nuclear ITS sequence variation; (b) date the divergence of the main clades; and (c) examine structural changes in the plastomes of the sampled species in the Salicaceae *s.l*.

## 2 MATERIALS AND METHODS

### 2.1 Plastome sequencing, assembly, and alignment

For each species (Supporting information Table S1), we extracted total DNA using the CTAB protocol (Allen, Floresvergara, Krasynanski, Kumar, & Thompson, 2006) from dried leaves preserved in silica gel. Illumina paired‐end libraries with an insert size of 500 base pairs (bp) were constructed and sequenced using the HiSeq X Ten System. At least two gigabases (Gb) of 2 × 150 bp short read data were generated for each sample. Reads with a Phred quality score <7 and more than 10% ambiguous nucleotides were filtered. The remaining reads were de novo assembled using the Velvet v1.2.07 (Zerbino & Birney, 2008) software. Contigs were connected into a linear sequence in Geneious v8.0.5 (Kearse et al., 2012) taking the *Populus tricocarpa* plastome as a reference. Annotation of plastomes was conducted using Plann v1.1 (Huang & Cronk, 2015). We extracted protein‐coding genes using customized Perl scripts. Alignment of chloroplast genes across all species was performed by PRANK v130410 (Löytynoja & Goldman, 2008). Poorly aligned regions were trimmed using Gblocks v0.91b (Castresana, 2000) with the option “−t=c” (i.e*.,* the type of sequence was set to codons). We discarded genes that were lost in at least one species and concatenated the aligned sequences into a super matrix.

### 2.2 ITS sequencing

The ITS was also sequenced for a few species (Supporting information Tables S2 and S3). We amplified this fragment on a GeneAmp PCR System 9700 thermal cycler (Applied Biosystems). The 25‐μl reaction mixture comprised 1 μl of template DNA, 2.5 μl of 10 × Taq Buffer (Mg^2+^ plus), 0.5 μl dNTP Mix (10 mM each), 0.5 μl of each primer, 1.25U of Taq DNA Polymerase. PCR products were confirmed on 1% agarose gels and then sent to Tsingke Biological Technology (Beijing, China) for sequencing. The ITS sequences were aligned using MEGA 7.0.18 (Kumar, Stecher, & Tamura, 2016).

### 2.3 Phylogenetic inference and divergence estimation

For plastid genes, we used RAxML v8.1.24 (Stamatakis, 2014) to conduct Maximum Likelihood (ML) analyses with the GTR+Γ model based on the 77 concatenated genes present in all 50 species (42 Salicaceae *s.l*. and eight outgroup species). The best‐scoring ML tree was obtained using the rapid hill‐climbing algorithm (i.e*.,* the option “‐f d”) with 1,000 bootstrap replicates. Due to the limited species sampling in the ITS dataset, we constructed an unrooted ML tree with 37 species (31 Salicaceae *s.l*. and 6 outgroup species) in MEGA 7.0.18 (Kumar et al., 2016), using complete deletion and the kimura 2‐parameter model. Bootstrap values were estimated with 1,000 random addition sequence replicates.

We estimated divergence times from the plastome dataset using an approximate likelihood method as implemented in MCMCtree (in PAML version 4) (Yang, 2007), with an independent relaxed‐clock and birth–death sampling (Rannala & Yang, 2007). Although the earliest fossils of the genera *Populus* and *Salix* based on leaves or leafy shoot with fruiting raceme could be dated back to the late Palaeocene and early Eocene, respectively (Collisin, 1992), accurate timings could not be determined. The split between *Populus* and *Salix* was therefore assigned a minimum age constraint of 48 Mya as has been used previously (Bell, Soltis, & Soltis, 2010). The root of the phylogeny after the exclusion of the more distant outgroups was restricted to a maximum age of 108 Mya based on the secondary age constraints described by Xi et al. (2012). The best‐fit GTR+Γ model was selected and the prior on the substitution rate (rgene) was modeled by a Γ distribution as Γ(2, 200, 1). We set parameters for the birth–death process with species sampling and σ2 values to 1 1 0.1 and G (1,10,1), respectively. We executed the MCMC runs for 2,000 generations as burn‐in and then sampled every 750 generations until a total of 20,000 samples had been obtained. We compared two MCMC runs for convergence using random seeds and obtained similar results.

## 3 RESULTS

### 3.1 Basic characteristics of plastomes

A total of 42 species of the family Salicaceae *s.l*. (Chase et al., 2002) were included in this study (Supporting information Table S1). Among these species, 28 were newly sequenced for plastomes, and these belonged to five genera (i.e*., Flacourtia*,* Itoa*,* Poliothyrsis*,* Populus*, and *Salix*). For the genus *Populus*, we sampled 25 of the 32 currently recognized species (Dickmann & Kuzovkina, 2008), representing all six sections. In addition, we sampled 13 species of *Salix*. The average length of the 42 aligned plastomes was 156.4 kilobases (kb), ranging from 155.0 kb (*Salix magnifica*) to 158.6 kb (*Populus adenopoda*). The average GC‐content was 36.7%, ranging from 36.5% (*P. ilicifolia*) to 37.0% (*S. interior*) (Supporting information Table S4).

### 3.2 Phylogenetic analyses of the Salicaceae *s.l*

To infer phylogenetic relationships of the 42 Salicaceae species, we also included 8 Malpighiales species, the plastomes of which are publicly available in GenBank. These species (*Chrysobalanus icaco*,* Couepia caryophylloides*,* Erythroxylum novogranatense*,* Gaulettia elata*,* Jatropha curcas*,* Parinari campestris*,* Ricinus communis*, and *Viola seoulensis*) were used as outgroups in our phylogenetic analyses. The final concatenated dataset included 77 plastid genes and 60,564 sites after trimming poorly aligned regions and gaps with missing genes.

**Figure 1:**
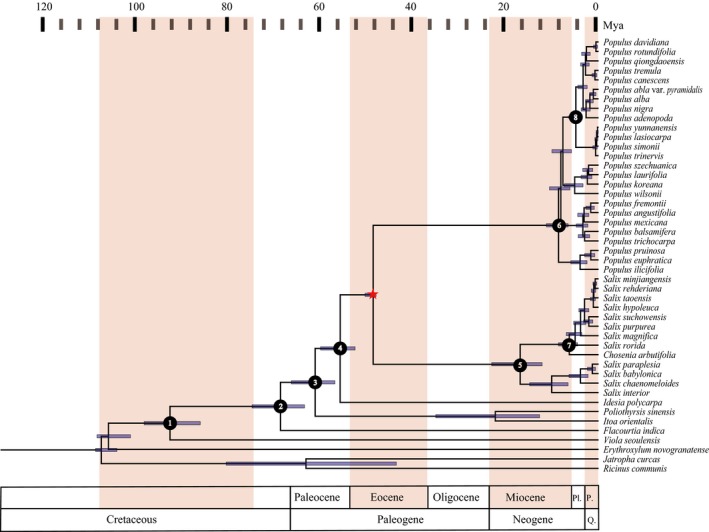
Phylogeny and clade divergence of Salicaceae *s.l*. and outgroups based on 77 plastome protein‐coding genes. Stars indicate fossil calibrations used in this analysis. Geological periods are marked with background colors. Mya: million years ago; P: Pleistocene; Pl: Pliocene; Q: Quaternary

The ML tree (Figure 1, Supporting information Figure S1 and S2) was derived from 1,000 bootstrap replicates. Phylogenetic relationships between the selected outgroups are largely consistent with previous studies (Wurdack & Davis, 2009; Xi et al., 2012) . Both Chrysobalanaceae (*Chrysobalanus*,* Couepia*,* Gaulettia*, and *Parinari*, sampled here) and Euphorbiaceae (*J*. *curcas* and *R*. *communis*) are monophyletic while *V. seoulensis* is placed as sister to Salicaceae *s.l*. The monophyly of 42 species of the Salicaceae *s.l*. was fully supported (100 bootstrap support value, BP; Figure 1). *Flacourtia* was identified as the basal clade; *Itoa* + *Poliothyrsis* and *Idesia* are successive sisters to Salicaceae *s.s*., which contains two genera *Populus* and *Salix*.

Five subclades were identified within the *Populus* clade (Supporting information Figure S1). The first subclade corresponds to sect. *Turanga*, in which *P*. *ilicifolia* is resolved (BP = 100%) as sister to *P*. *euphratica* plus *P*. *pruinosa*. The second subclade includes *P*. *angustifolia*,* P*. *balsamifera*, and *P*. *trichocarpa* of sect. *Tacamahaca, Populus mexicana* of sect. *Abaso* and *P*. *fremontii* of sect. *Aigeiros*. Here, *P*. *fremontii* is placed as sister to *P*. *angustifolia* with BP = 100%. Within the third subclade, three species *P*. *koreana*,* P*. *laurifolia*, and *P*. *szechuanica* of sect. *Tacamahaca* were monophyletic (BP = 100%) and placed as sister to *P*. *wilsonii* (sect. *Leucoides*). The fourth subclade contains *P. trinervis, P*. *simonii*,* P*. *yunnanensis* of sect. *Tacamahaca* (as monophyletic) and *P*. *lasiocarpa* of sect. *Leucoides*. The fifth subclade includes *P*. *adenopoda*,* P*. *alba*,* P*. *alba* var. *pyramidalis*,* P*.* *× *canescens*,* P*. *davidiana*,* P*.* qiongdaoensis*,* P*. *rotundifolia*, and *P*.* tremula* of sect. *Populus* and *P*. *nigra* of sect. *Aigeiros*. Here, *P*.* *× *canescens*, a well‐known hybrid between *P*. *alba* and *P*.* tremula*, is placed as sister to one of its parental taxa *P*.* tremula* with BP = 100%, while the other parent *P*. *alba* was resolved to be sister to *P*. *nigra*. The successive divergences between the second, the third, and the other subclades received lower support (BP = 75% and BP = 81%, respectively). Two well‐supported subclades were identified within the *Salix* clade.

We further constructed the phylogenetic relationships of 31 species of Salicaceae *s.l*. based on the nuclear ITS sequence variations (Supporting information Figure S3). Like plastome phylogeny, the monophylies of Salicaceae *s.l*. and the genus *Populus* received high support (BP = 100%). Two major subclades were recovered in the genus *Salix*. Similarly, *Flacourtia* was also identified as the basal clade of Salicaceae *s.l*. while the *Itoa, Poliothyrsis*, and *Idesia* clade was identified as sister to Salicaceae *s.s*. with poor support. Within the *Populus* clade, six species of sect. *Populus* clustered into a monophyletic subclade with low support (BP = 68%). The relationships between the subclade and other species remained poorly supported.

### 3.3 Divergence estimates of main clades and subclades

We estimated divergence timescales of the major clades within the Salicaceae *s.l*. according to the calibrations of the gene tree constructed on the basis of 77 plastid genes. The family diverged from the sister outgroup 92 Mya (Figure 1 and Table 1). The basal *Flacourtia* was estimated to diverge from other clades around 69 Mya while the next two successive clades (*Itoa* + *Poliothyrsis*) and *Idesia* were estimated to have originated around 61 and 56 Mya, respectively. Two major subclades of the genus *Salix s.l*. diverged around 17 Mya. The crown ages of all subclades in the genera *Populus* and *Salix* were dated mainly within the Pliocene, suggesting that the numerous species of these two genera originally diversified within the recent past (6 Mya) (Figure 1 and Table 1).

**Table 1:** Estimated ages for major Salicaceae *sensu lato* subclades

Subclade name^a^	Mean age (Mya)	95% highest posterior density interval (HPD)
Subclade 1	92.5	86.0–98.1
Subclade 2	68.7	63.2–74.9
Subclade 3	61.2	56.9–66.3
Subclade 4	55.8	52.6–60.0
Subclade 5	16.9	12.2–23.1
Subclade 6	8.6	6.57–11.28
Subclade 7	8.1	6.19–10.60
Subclade 8	4.9	3.61–6.61

*Note*

a Subclades are labeled in Figure 1.

### 3.4 Gene loss

**Figure 2:**
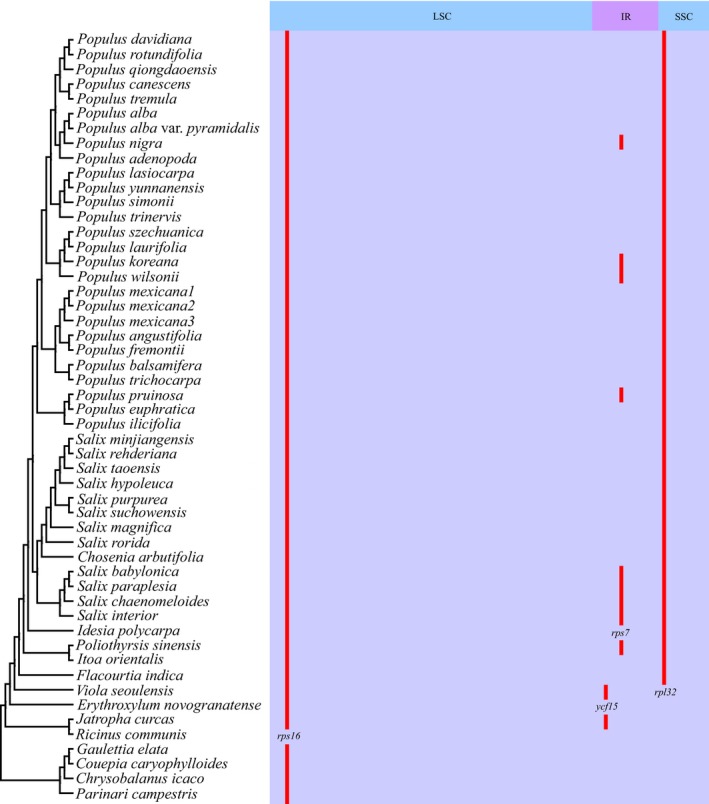
Loss of chloroplast protein‐coding genes across Salicaceae *s.l*. and outgroups as indicated in Figure 1. Gray and red boxes indicate intact and possible pseudogenized genes, respectively. IR: inverted repeat; LSC: large single‐copy region; SSC: small single‐copy region

A total of 77 major protein coding genes are present in all plastomes as in most angiosperms (Figure 2). The *rpl32* gene was absent from all sampled species of the Salicaceae *s.l*., but present in all sampled outgroups. However, the *rps16* gene was absent not only in the Salicaceae *s.l*., but also in all outgroups except for *R*. *communis*. The *rps7* gene became a pseudogene in the plastomes of the Salicaceae *s.l*. four separate times, based on the fact that the species with this pseudogene did not cluster into one monophyletic group.

## 4 DISCUSSION

Salicaceae *s.l*. was confirmed here as a monophyletic group, based not only on phylogenetic analyses of the plastome sequences, but also from the gene content. We found that the absence of the *rpl32* gene is a potential synapomorphy for this family in the broad sense. We obtained well‐resolved phylogenetic relationships between most sampled species, clades and subclades of the family. The resolution and support between *Flacourtia*,* Itoa*,* Poliothyrsis*,* Idesia*, and Salicaceae *s.s*. were greatly improved compared with using only nuclear ITS sequence variations (Supporting information Figure S3), but consistent with those constructed based on only 13 genes (Wurdack & Davis, 2009; Xi et al., 2012). This well‐resolved plastome phylogeny will be very useful for constructing relationships within Salicaceae *s.l*. if even more genera of the family could be sampled.

Within the Salicaceae *s.s*., both *Salix* and *Populus* were robustly supported as monophyletic clades. As found before, two main subclades were identified for *Salix* (Chen et al., 2010; Wu et al., 2015). In addition, we also found interspecific relationships for the sampled willow species in each subclade inferred from plastomes that are not consistent with those phylogenies reported before based on ITS and limited chloroplast DNA (Chen et al., 2010; Wu et al., 2015). More inconsistences were found for the genus *Populus*. Five well‐supported subclades were recovered (Figure 1). However, except for sect. *Turanga*, none of the other sections defined before based on morphological traits (Eckenwalder, 1996; Fang et al., 1999) were supported (Supporting information Figure S1 and S2). The interrelationships between the five subclades received medium (BP = 71%) to high (BP = 100%) support. The recovered subclades and their interrelationships were strongly supported, but distinctly different from those based on nuclear ITS (Supporting information Figure S3) or limited chloroplast DNA (Cervera et al., 2005; Hamzeh et al., 2006; Wan et al., 2013). Two nonexclusive factors may explain these conflicts in both willows and poplars. First, hybridizations are extremely common between different species and sections because of the incomplete reproductive isolation in both genera (6, 11, 20). For example, most species of different sections can be hybridized in the genus *Populus* (except between sect. *Turanga* and other sections). These hybridizations lead to introgressions of the maternally inherited plastome (Currat, Ruedi, Petit, & Excoffier, 2008; Du, Petit, & Liu, 2009). Furthermore, such hybridizations can lead to the random concerted evolution of ITS sequences with multiple copies from one of the two parents (Koch, Dobes, & Mitchell‐Olds, 2003; Wendel, Schnabel, & Seelanan, 1995). Second, incomplete lineage sorting is likely to have persisted widely for these long‐generation trees or shrubs. Even assuming a simple and ideal allopatric speciation, a long time (9–12 generations) is required to sort two incipient species into reciprocally monophyletic clades at most loci according to the pure drift hypothesis (Hudson & Coyne, 2002). Genetic diversity is, therefore, commonly shared between recently diverged species with a long‐generation life. When different individuals or loci are sampled, conflicting phylogenies will appear. However, it is difficult to identify whether incomplete lineage sorting or gene flow caused by interspecific hybridization produced the conflicting phylogenies recovered here for both willows and poplars. Further studies based on nuclear genomic data, especially at the population level, are needed to clarify these respective contributions and construct species trees and evolutionary histories of both genera.

The high conservation and stable alignment of the 77 plastid genes allowed us to calibrate the divergences and origins of the main clades in the Salicaceae *s.l*. (Figure 1). Because accurate ages of any of the fossils found for this family remain difficult to determine, we used two tentative calibrations to estimate diversification. All estimated ages should be used with caution. We found that the whole family diverged from the sister family around 92 Mya and the three successive clades within the family diverged 69, 61, and 55 Mya, suggesting relatively late clade diversifications. Specifically, most species diversifications based on these plastid genes within the main subclades of both *Populus* and *Salix* were estimated to have occurred in the recent past, mostly after 6 Mya, despite the fact that numerous species are currently acknowledged in both genera, especially in the genus *Salix*. This may partly explain the widespread hybridization between these young species even from different sections (Eckenwalder, 1996; Fang et al., 1999), resulting from incomplete reproductive isolation. The divergence timescales estimated here for major clades and subclades, will provide a basic timescale to take diverse studies of this model family forward.

## CONFLICTS OF INTEREST

The authors declare no conflict of interest.

## AUTHOR CONTRIBUTIONS

Tao Ma conceived and designed the experiments; Lei Zhang, Zhenxiang Xi, Mingcheng Wang, Xinyi Guo undertook the experiments and analyses. Lei Zhang and Tao Ma wrote the manuscript.
