# Supplementary material for: Plastome phylogeny and lineage diversification of Salicaceae with focus on poplars and willows
Source: Ecol Evol. 2018 Jul 13;8(16):7817–23. doi: 10.1002/ece3.4261 (PMC6145263; doi:10.1002/ece3.4261)
Supplement: Supplementary file 4 [file ECE3-8-7817-s004.doc]

**TABLE** **1** Estimated ages for major Salicaceae *sensu lato* subclades

| **Subclade name^*^** | **Mean age (Mya)** | **95% highest posterior density interval (HPD)** |
| --- | --- | --- |
| Subclade 1 | 92.5 | 86.0-98.1 |
| Subclade 2 | 68.7 | 63.2-74.9 |
| Subclade 3 | 61.2 | 56.9-66.3 |
| Subclade 4 | 55.8 | 52.6-60.0 |
| Subclade 5 | 16.9 | 12.2-23.1 |
| Subclade 6 | 8.6 | 6.57-11.28 |
| Subclade 7 | 8.1 | 6.19-10.60 |
| Subclade 8 | 4.9 | 3.61-6.61 |

^*^Subclades are labeled in Figure 1

**TABLE** **S1** Species and data description of plastomes used in this study.

| **Source** | **Taxon** | **Genbank accession** | **Collection number** | **Collector** | **Collection date** | **Herbarium deposit****^*^** |
| --- | --- | --- | --- | --- | --- | --- |
| Assembled in our lab | *Chosenia arbutifolia* (Pall.) A. Skv. | MG262340 | LJQ-NEC-2016-001 | Lei Zhang | Jun-2016 | SZ |
|  | *Flacourtia indica* (N.L.Burm.) Merr. | MG262341 | LJQ-ZL-2015-059 | Lei Zhang | Jun-2015 | SZ |
|  | *Itoa orientalis* Hemsl. | MG262342 | LJQ-ZL-2015-060 | Lei Zhang | Jun-2015 | SZ |
|  | *Poliothyrsis sinensis* Oliv. | MG262343 | LJQ-ZL-2015-061 | Lei Zhang | Jun-2015 | SZ |
|  | *Populus adenopoda* Maxim. | KX425622 | MaoKS-CX-2014-307 | Kangshan Mao | Jun-2014 | SZ |
|  | *Populus angustifolia* E. James | MG262345 | LJQ-ZL-2015-062 | Lei Zhang | Jun-2015 | SZ |
|  | *Populus canescens* (Ait.) Smith. | MG262346 | LJQ-NEC-2015-059 | Lei Zhang | Jul-2015 | SZ |
|  | *Populus davidiana* Dode | MG262347 | LiuJQ-MZL-2013-327 | Kangshan Mao | Jun-2013 | SZ |
|  | *Populus koreana* Rehd. | MG262348 | LJQ-NEC-2015-017 | Lei Zhang | Jul-2015 | SZ |
|  | *Populus lasiocarpa* Oliv. | MG262349 | LJQ-SHX-2015-012 | Lei Zhang | Jun-2015 | SZ |
|  | *Populus laurifolia* Ledeb. | MG262350 | LJQ-ZL-2015-063 | Lei Zhang | Jun-2015 | SZ |
|  | *Populus mexicana* Wesmael 1 | MG262351 | PM11_S6 | Steve | - | - |
|  | *Populus mexicana* Wesmael 2 | MG262352 | PM11_S6 | Steve | - | - |
|  | *Populus mexicana* Wesmael 3 | MG262353 | PM11_S6 | Steve | - | - |
|  | *Populus nigra* L. | MG262354 | LiuJQ-XJ-2011-047 | Jianju Feng | Agu-2011 | SZ |
|  | *Populus pruinosa* Schrenk | MG262355 | Liuiq-fjj-0216-024 | Jianju Feng | Agu-2011 | SZ |
|  | *Populus alba* f. *pyramidalis* (Bge.) Dip. | MG262344 | LiuJQ-fjj-0267-119 | Jianju Feng | Agu-2011 | SZ |
|  | *Populus simonii* Carr. | MG262356 | MaoKS-CX-2014-019 | Kangshan Mao | Jun-2014 | SZ |
|  | *Populus szechuanica* C. K.Schneid. | MG262357 | MaoKS-CX-2014-048 | Kangshan Mao | Jun-2014 | SZ |
|  | *Populus trinervis* C. Wang & S. L. Tung | MG262358 | MaoKS-CX-2014-050 | Kangshan Mao | Jun-2014 | SZ |
|  | *Populus wilsonii* C. K. Schneid. | MG262359 | LJQ-SHX-2015-016 | Lei Zhang | Jun-2015 | SZ |
|  | *Populus yunnanensis* Dode | MG262360 | MaoKS-CX-2014-270 | Lei Zhang | Jun-2014 | SZ |
|  | *Salix babylonica* L. | MG262361 | ZL-SCU-2017-001 | Lei Zhang | Apr-2017 | SZ |
|  | *Salix chaenomeloides* Kimura | MG262362 | LJQ-SHX-2016-001 | Lei Zhang | May-2016 | SZ |
|  | *Salix hypoleuca* Seem. ex Diel. | MG262363 | LJQ-XZZ-017 | Lei Zhang | Apr-2016 | SZ |
|  | *Salix magnifica* Hemsl. | MG262364 | ZL-006 | Lei Zhang | Agu-2016 | SZ |
|  | *Salix minjiangensis* N. Chao | MG262365 | ZL-015 | Lei Zhang | Agu-2016 | SZ |
|  | *Salix paraplesia* Schneid. | MG262366 | ZR-013 | Lei Zhang | Agu-2016 | SZ |
|  | *Salix rehderiana* C. K.Schneid. | MG262367 | LJQ-XZZ-008 | Lei Zhang | May-2016 | SZ |
|  | *Salix rorida* Laksch. | MG262368 | LJQ-NEC-2015-020 | Lei Zhang | Jul-2015 | SZ |
|  | *Salix* *taoensis* Goerz ex Rehd. & Kobus. | MG262369 | ZR-014 | Lei Zhang | Agu-2016 | SZ |
| Downloaded from Genbank | *Chrysobalanus icaco* L. | NC_024061 | - | - | - | - |
|  | *Couepia caryophylloides* Benoist | NC_030547 | - | - | - | - |
|  | *Erythroxylum novogranatense* | NC_030601 | - | - | - | - |
|  | *Gaulettia elata* (Ducke) Sothers | NC_030559 | - | - | - | - |
|  | *Idesia polycarpa* Maxim. | NC_032060 | - | - | - | - |
|  | *Jatropha curcas* L. | NC_012224 | - | - | - | - |
|  | *Parinari campestris* Aubl. | NC_024067 | - | - | - | - |
|  | *Populus alba* L. | NC_008235 | - | - | - | - |
|  | *Populus balsamifera L.* | NC_024735 | - | - | - | - |
|  | *Populus euphratica* Oliv. | NC_024747 | - | - | - | - |
|  | *Populus fremontii* S. Watson | NC_024734 | - | - | - | - |
|  | *Populus ilicifolia* (Engl.) Roul. | KX421095 | - | - | - | - |
|  | *Populus qiongdaoensis* T. Hong & P. Luo | KX534066 | - | - | - | - |
|  | *Populus rotundifolia* var. *duclouxiana* (Dode) Gomb. | KX425853 | - | - | - | - |
|  | *Populus tremula* L. | NC_027425 | - | - | - | - |
|  | *Populus trichocarpa* Torr. | NC_009143 | - | - | - | - |
|  | *Ricinus communis* L. | NC_016736 | - | - | - | - |
|  | *Salix interior* Rowl. | NC_024681 | - | - | - | - |
|  | *Salix purpurea* L. | NC_026722 | - | - | - | - |
|  | *Salix suchowensis* W. C. Cheng ex G. Zhu | NC_026462 | - | - | - | - |
|  | *Viola seoulensis* Nakai | KP749924 | - | - | - | - |

^*^SZ：Herbarium of Sichuan University

**TABLE S2** Details of the ITS used in this study.

| **Regions** | **Primer pairs** | **Sequence 5'-3'** | **Thermocycle profile** |
| --- | --- | --- | --- |
| ITS | ITS1 | AGAAGTCGTAACAAGGTTTCCGTAGG | 95℃3min；[35cycles：95℃15s；54℃15s；72℃60s]；72℃5min |
|  | ITS4 | TCCTCCGCTTATTGATATGC |  |

**TABLE S3** Species and data description of ITS used in this study.

| **Source** | **Taxon** | **Genbank accession** | **Collection number** | **Collector** | **Collection date** | **Herbarium deposit^*^** |
| --- | --- | --- | --- | --- | --- | --- |
| Obtained by our lab | *Poliothyrsis sinensis* Oliv. | MG015700 | LJQ-ZL-2015-061 | Lei Zhang | Jun-2015 | SZ |
|  | *Populus adenopoda* Maxim. | MG015701 | MaoKS-CX-2014-307 | Kangshan Mao | Jun-2014 | SZ |
|  | *Populus canescens* (Ait.) Smith. | MG015702 | LJQ-NEC-2015-059 | Lei Zhang | Jul-2015 | SZ |
|  | *Populus koreana* Rehd. | MG015703 | LJQ-NEC-2015-017 | Lei Zhang | Jul-2015 | SZ |
|  | *Populus tremula* L. | MG015704 | LiuJQ-XJ-2011-019 | Jianju Feng | Agu-2011 | SZ |
|  | *Populus wilsonii* C. K. Schneid. | MG015705 | LJQ-SHX-2015-016 | Lei Zhang | Jun-2015 | SZ |
|  | *Salix magnifica* Hemsl. | MG015706 | ZL-006 | Lei Zhang | Agu-2016 | SZ |
|  | *Salix minjiangensis* N. Chao | MG015707 | ZL-015 | Lei Zhang | Agu-2016 | SZ |
|  | *Salix rehderiana* C. K.Schneid. | MG015708 | LJQ-XZZ-008 | Lei Zhang | May-2016 | SZ |
|  | *Salix taoensis* Goerz ex Rehd. & Kobus. | MG015709 | ZR-014 | Lei Zhang | Agu-2016 | SZ |
| Downloaded from Genbank | *Chosenia arbutifolia* (Pall.) A. Skv. | EF060366 | - | - | - | - |
|  | *Chrysobalanus icaco* L. | JQ898993 | - | - | - | - |
|  | *Couepia caryophylloides* Benoist | JQ899002 | - | - | - | - |
|  | *Flacourtia indica* (N.L.Burm.) Merr. | DQ521289 | - | - | - | - |
|  | *Idesia polycarpa* Maxim. | AJ006441 | - | - | - | - |
|  | *Itoa orientalis* Hemsl. | KC999840 | - | - | - | - |
|  | *Jatropha curcas* L. | KP191021 | - | - | - | - |
|  | *Parinari campestris* Aubl. | JQ898976 | - | - | - | - |
|  | *Populus alba* L. | JQ898650 | - | - | - | - |
|  | *Populus davidiana* Dode | KC485087 | - | - | - | - |
|  | *Populus euphratica* Oliv. | KC485090 | - | - | - | - |
|  | *Populus lasiocarpa* Oliv. | JQ898628 | - | - | - | - |
|  | *Populus laurifolia* Ledeb. | KC485094 | - | - | - | - |
|  | *Populus nigra* L. | KC485095 | - | - | - | - |
|  | *Populus pruinosa* Schrenk | KC485100 | - | - | - | - |
|  | *Populus rotundifolia* var. *duclouxiana* (Dode) Gomb. | KC485102 | - | - | - | - |
|  | *Populus simonii* Carr. | KC485103 | - | - | - | - |
|  | *Populus szechuanica* C. K.Schneid. | KC485104 | - | - | - | - |
|  | *Populus trichocarpa* Torr. | JQ898636 | - | - | - | - |
|  | *Populus yunnanensis* Dode | JQ898623 | - | - | - | - |
|  | *Ricinus communis* L. | KJ130047 | - | - | - | - |
|  | *Salix babylonica* L. | KX008770 | - | - | - | - |
|  | *Salix chaenomeloides* Kimura | KC415504 | - | - | - | - |
|  | *Salix paraplesia* Schneid. | KC415528 | - | - | - | - |
|  | *Salix purpurea* Linn. | GU556175 | - | - | - | - |
|  | *Salix interior* Rowl. | KX008852 | - | - | - | - |
|  | *Viola seoulensis* Nakai | AY928301 | - | - | - | - |

^*^SZ：Herbarium of Sichuan University

**TABLE S4** Comparison of sequence length and GC content of plastomes across Salicaceae *s.l.* and outgroups.

| **Source** | **Taxon** | **Full Sequence** | | **LSC_region** | | **SSC_region** | | **IR_region** | |
| --- | --- | --- | --- | --- | --- | --- | --- | --- | --- |
|  |  | **Length** | **GC_content** | **LSC_length** | **GC_content** | **SSC_length** | **GC_content** | **IR_length** | **GC_content** |
| This study | *Chosenia arbutifolia* (Pall.) A. Skv. | 155055 | 36.7% | 83989 | 34.5% | 16162 | 31.0% | 27453 | 41.2% |
|  | *Flacourtia indica* (N.L.Burm.) Merr. | 156542 | 36.7% | 84488 | 34.5% | 16484 | 30.1% | 27784 | 41.9% |
|  | *Itoa orientalis* Hemsl. | 156682 | 36.8% | 84479 | 34.7% | 16537 | 30.6% | 27833 | 41.8% |
|  | *Poliothyrsis sinensis* Oliv. | 156680 | 36.8% | 84793 | 34.7% | 16544 | 30.6% | 27641 | 41.9% |
|  | *Populus adenopoda* Maxim. | 158591 | 36.7% | 84633 | 34.5% | 18622 | 30.9% | 27668 | 42.0% |
|  | *Populus angustifolia* E. James | 156698 | 36.7% | 84771 | 34.5% | 16529 | 30.6% | 27699 | 34.5% |
|  | *Populus canescens* (Ait.) Smith. | 157442 | 36.7% | 85364 | 34.4% | 16784 | 30.6% | 27647 | 41.9% |
|  | *Populus davidiana* Dode | 157403 | 36.6% | 85177 | 34.5% | 16814 | 30.5% | 27706 | 41.9% |
|  | *Populus ilicifolia* (Engl.) Roul. | 158017 | 36.5% | 85926 | 34.3% | 16533 | 30.6% | 27778 | 41.8% |
|  | *Populus koreana* Rehd. | 156800 | 36.7% | 85332 | 34.5% | 16602 | 30.7% | 27433 | 42.0% |
|  | *Populus lasiocarpa* Oliv. | 156491 | 36.7% | 84771 | 34.5% | 16480 | 30.5% | 27620 | 42.0% |
|  | *Populus laurifolia* Ledeb. | 157168 | 36.6% | 85071 | 34.4% | 16683 | 30.5% | 27707 | 41.9% |
|  | *Populus mexicana* Wesmael 1 | 156947 | 36.7% | 85223 | 34.5% | 16563 | 30.5% | 27579 | 41.9% |
|  | *Populus mexicana* Wesmael 2 | 157080 | 36.7% | 85360 | 34.5% | 16566 | 30.5% | 27577 | 41.9% |
|  | *Populus mexicana* Wesmael 3 | 157243 | 36.7% | 85223 | 34.6% | 16563 | 30.5% | 27577 | 41.9% |
|  | *Populus nigra* L. | 155096 | 36.8% | 83672 | 34.7% | 16564 | 30.5% | 27430 | 42.0% |
|  | *Populus pruinosa* Schrenk | 158551 | 36.6% | 85761 | 34.3% | 16540 | 30.7% | 28125 | 41.8% |
|  | *Populus alba* f. *pyramidalis* (Bge.) Dip. | 156342 | 36.8% | 84480 | 34.6% | 16538 | 30.5% | 27662 | 42.0% |
|  | *Populus qiongdaoensis* T. Hong & P. Luo | 156074 | 36.7% | 84466 | 34.6% | 16511 | 30.5% | 27548 | 42.0% |
|  | *Populus rotundifolia* var. *duclouxiana* (Dode) Gomb. | 155373 | 36.8% | 84603 | 34.5% | 16806 | 30.5% | 26982 | 42.3% |
|  | *Populus simonii* Carr. | 156637 | 36.7% | 84836 | 34.5% | 16486 | 30.5% | 27658 | 42.0% |
|  | *Populus szechuanica* C. K.Schneid. | 156742 | 36.7% | 84843 | 34.5% | 16499 | 30.7% | 27700 | 41.9% |
|  | *Populus trinervis* C. Wang & S. L. Tung | 156409 | 36.7% | 84802 | 34.5% | 16503 | 30.5% | 27552 | 42.0% |
|  | *Populus wilsonii* C. K. Schneid. | 157095 | 36.7% | 85010 | 34.5% | 16619 | 30.6% | 27733 | 41.8% |
|  | *Populus yunnanensis* Dode | 156445 | 36.7% | 84691 | 34.5% | 16438 | 30.6% | 27658 | 42.0% |
|  | *Salix babylonica* L. | 155697 | 36.6% | 84467 | 34.4% | 16320 | 31.0% | 27455 | 41.7% |
|  | *Salix chaenomeloides* Kimura | 156154 | 36.7% | 85278 | 34.4% | 16302 | 31.0% | 27287 | 42.0% |
|  | *Salix hypoleuca* Seem. ex Diel. | 155126 | 36.7% | 83975 | 34.5% | 16235 | 31.0% | 27458 | 41.9% |
|  | *Salix magnifica* Hemsl. | 154977 | 36.7% | 83857 | 34.5% | 16204 | 31.0% | 27458 | 41.9% |
|  | *Salix minjiangensis* N. Chao | 155038 | 36.7% | 83925 | 34.5% | 16215 | 31.0% | 27449 | 41.8% |
|  | *Salix paraplesia* Schneid. | 155553 | 36.7% | 84340 | 34.5% | 16303 | 31.0% | 27455 | 41.7% |
|  | *Salix rehderiana* C. K.Schneid. | 155051 | 36.7% | 83914 | 34.5% | 16219 | 31.0% | 27459 | 41.9% |
|  | *Salix rorida* Laksch. | 155144 | 36.7% | 84011 | 34.5% | 16213 | 31.0% | 27460 | 41.9% |
|  | *Salix taoensis* Goerz ex Rehd. & Kobus. | 155085 | 36.7% | 83947 | 34.5% | 16220 | 31.0% | 27459 | 41.9% |
| NCBI | *Chrysobalanus icaco* L. | 162775 | 36.2% | 89187 | 33.9% | 19818 | 29.5% | 26885 | 42.9% |
|  | *Couepia caryophylloides* Benoist | 161887 | 36.3% | 88935 | 33.9% | 19426 | 29.9% | 26216 | 42.5% |
|  | *Erythroxylum novogranatense* | 163937 | 35.9% | 91383 | 33.2% | 18138 | 30.2% | 27208 | 42.2% |
|  | *Gaulettia elata* (Ducke) Sothers | 162303 | 36.3% | 88846 | 33.9% | 19703 | 29.8% | 26877 | 42.6% |
|  | *Idesia polycarpa* Maxim. | 157017 | 36.7% | 84787 | 34.5% | 16512 | 20.6% | 27859 | 41.9% |
|  | *Jatropha curcas* L. | 163856 | 35.4% | 91731 | 32.9% | 17849 | 28.9% | 27138 | 42.2% |
|  | *Parinari campestris* Aubl. | 162637 | 36.2% | 89212 | 33.9% | 19713 | 29.7% | 26856 | 42.5% |
|  | *Populus alba* L. | 156505 | 36.7% | 84618 | 34.6% | 16567 | 30.5% | 27660 | 41.9% |
|  | *Populus balsamifera L.* | 157094 | 36.7% | 84921 | 34.5% | 16499 | 30.4% | 27846 | 41.8% |
|  | *Populus euphratica* Oliv. | 156766 | 36.7% | 84887 | 34.5% | 16589 | 30.6% | 27646 | 41.9% |
|  | *Populus fremontii* S. Watson | 157446 | 36.7% | 85454 | 34.5% | 16316 | 30.5% | 27838 | 41.9% |
|  | *Populus tremula* L. | 156067 | 36.8% | 84377 | 34.6% | 16490 | 30.6% | 27600 | 42.0% |
|  | *Populus trichocarpa* Torr. | 157033 | 36.7% | 85129 | 34.5% | 16600 | 30.5% | 27652 | 41.9% |
|  | *Ricinus communis* L. | 163161 | 35.7% | 89651 | 33.3% | 18816 | 29.5% | 27347 | 41.9% |
|  | *Salix interior* Rowl. | 156620 | 37.0% | 85979 | 34.9% | 16306 | 30.8% | 27167 | 42.1% |
|  | *Salix purpurea* L. | 155590 | 36.7% | 84452 | 34.4% | 16621 | 31.0% | 27458 | 41.9% |
|  | *Salix suchowensis* W. C. Cheng ex G. Zhu | 155214 | 36.7% | 84077 | 34.3% | 16220 | 31.0% | 27459 | 41.9% |
|  | *Viola seoulensis* Nakai | 156507 | 36.3% | 85691 | 33.8% | 18010 | 29.6% | 26403 | 42.6% |
